# Cavernous Transformation of Portal Vein in the Setting of Protein C and Anti-thrombin III Deficiency

**DOI:** 10.7759/cureus.5779

**Published:** 2019-09-27

**Authors:** Mahwish Nasim, Bushra Majid, Faryal Tahir, Zainab Majid, Iqra Irfan

**Affiliations:** 1 Pediatrics, Dow University of Health Sciences, Karachi, PAK; 2 Internal Medicine, Dow University of Health Sciences, Karachi, PAK

**Keywords:** cavernous transformation, portal vein, portal hypertension, protein c deficiency, anti thrombin iii deficiency, pediatric, transjugular intrahepatic portosystemic shunt

## Abstract

Cavernous transformation of the portal vein (CTPV), also known as portal cavernoma, is a sequelae of thrombosis in the portal vein causing its occlusion and portal hypertension. The etiology, however, remains unknown. Gastroesophageal variceal bleeding, splenomegaly, portosystemic collaterals, and ultimate hematologic abnormalities are among the prominent clinical features. Among the causes, predisposing an individual to CTPV is natural anticoagulant protein C and antithrombin III deficiencies. Determination of the etiology of CTPV may also give a direction toward the management plan to not only relieve the patient of the already developed complications but also to treat the primary cause of the pathology

We discuss a case of a nine-year-old male child diagnosed as CTPV secondary to protein C and antithrombin III deficiency who was treated symptomatically for anemia and varices and was referred for transjugular intrahepatic portosystemic shunt (TIPS).

## Introduction

Cavernous transformation of the portal vein (CTPV) is a rare condition with numerous etiologies and a vast array of clinical presentations [1]. It is usually secondary to long-standing portal vein thrombosis (PVT) that causes portal hypertension and occlusion of the portal vein, consequently leading to the development of multiple small dilated blood vessels in and around the native portal vein [2-3]. The commonly affected population includes patients with healthy livers with prolonged non-cirrhotic and non-tumoral PVT. Yet, the etiology is unknown [4]. Gastroesophageal variceal bleeding, splenomegaly, portosystemic collaterals, and ultimate hematologic abnormalities are among the prominent clinical features. Confirmation of diagnosis depends upon abdominal ultrasonography, color Doppler ultrasound (US), computed tomography (CT) angiography, and magnetic resonance imaging (MRI).

PVT is referred to as complete or partial occlusion of blood flow in the portal vein preceded by a thrombus in its lumen [5]. The first case of PVT showing splenomegaly and ascites along with variceal dilation was reported in 1868 by Balfour and Stewart [6]. Acute PVT presents with intestinal congestion and ischemia, abdominal pain and distension with diarrhea, bleeding per rectum, splenomegaly, fever, nausea, vomiting, anorexia, lactic acidosis, and sepsis [6]. On the other hand, chronic PVT, more challenging to diagnose, usually runs an asymptomatic course or may present with splenomegaly, varices, and pancytopenia. Rarely, ascites can also be a feature of chronic PVT [6]. In all cases of PVT, patients should always be tested for an underlying thrombophilic condition [7]. Natural anticoagulants, namely protein C and antithrombin III deficiencies, are among the common hereditary thrombophilias, known to predispose for the development of PVT. Due to the increased demand and improvement of non-invasive imaging techniques for the diagnostic assessment of abdominal pain, portomesenteric venous obstruction is now a commonly recognized culprit [5-6,8-9].

We report a case of a nine-year-old male child diagnosed as CPTV secondary to protein C and antithrombin III deficiency who presented with generalized weakness, pancytopenia, portal vein thrombosis, splenomegaly, and perisplenic and splenorenal varices.

## Case presentation

A nine-year-old male child presented to the pediatric department of Dr. Ruth KM Pfau Civil Hospital, Karachi (CHK), with a complaint of generalized weakness for four months, which was associated with decreased exercise tolerance. The patient showed normal development according to age. There was no history of respiratory distress, bone pain, altered level of consciousness, jaundice, rash, upper or lower gastrointestinal blood loss, and fits or contraction of any infectious disease contact like Tuberculosis. There was no history of umbilical catheterization during neonatal life. Family history was also negative for similar illness.

On clinical examination, we found a vitally stable, pale-looking, anicteric male child, with average built and mildly distended abdomen. Abdominal examination revealed a scaphoid, non-tender abdomen with centrally placed umbilicus and no visible veins. A massive, firm, splenomegaly was present, 9 cm below the left costal margin. Ascites could not be appreciated, and the rest of the examination was normal.

**Figure 1:**
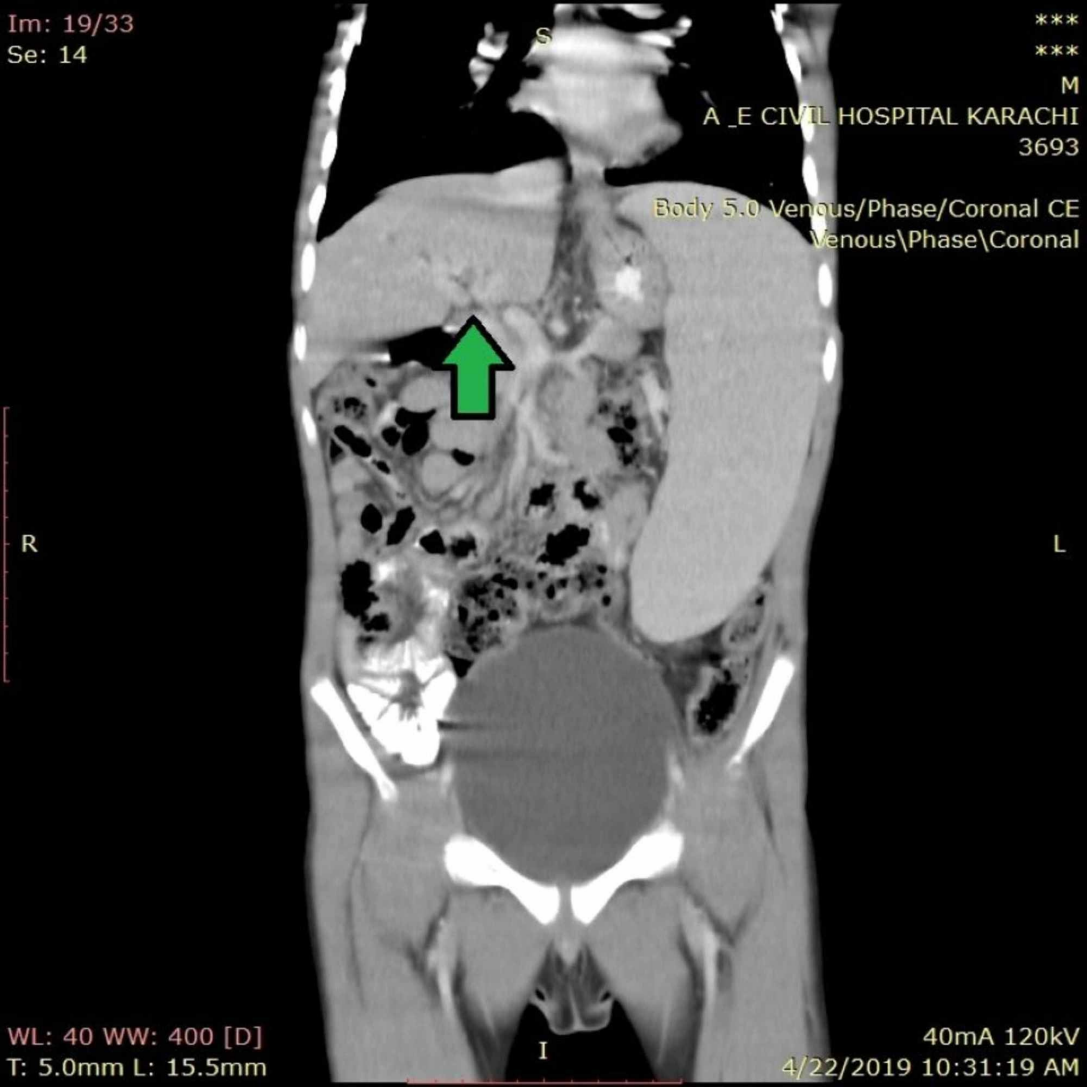
Coronal section of CT abdomen of patient showing cavernous transformation of portal vein at porta hepatis CT, computed tomography

**Figure 2:**
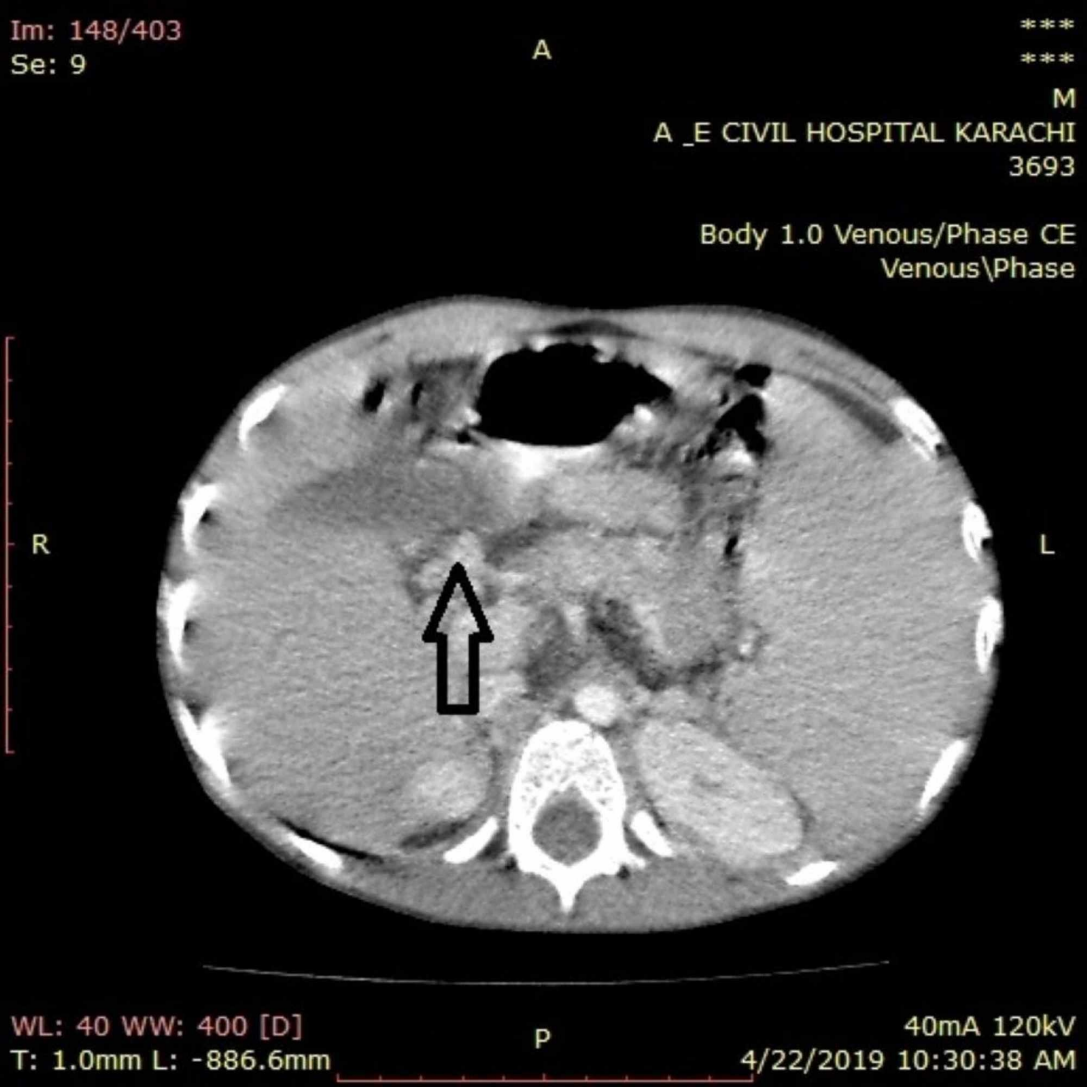
Axial section of CT abdomen showing cavernous transformation of portal vein CT, computed tomography

Initial lab reports showed iron-deficiency anemia (iron = 34 μg/dL, ferritin = 7.59 ng/dL, total iron-binding capacity = 328 μg/dL) with thrombocytopenia (platelets = 54,000/μL), and neutropenia (neutrophils = 55%). Peripheral smear of blood showed anisocytosis and microchromic anemia with a possibility of nutritional anemia. Liver function test showed bilirubin 0.5 mg/dl, serum glutamic pyruvic transaminase 25 IU/L, serum glutamic oxaloacetic transaminase 40 IU/L, serum alkaline phosphatase 244 IU/L, serum albumin 4.1 g/dl, prothrombin time 11.9 s, and international normalized ratio (INR) of 1.12. Viral markers for Hepatitis B and Hepatitis C were tested using enzyme-linked immunosorbent assay (ELISA) and came out to be negative. Ultrasound abdomen showed enlarged spleen measuring 15.0 cm with homogeneous parenchyma. The splenic vein was dilated measuring 0.9 cm with splenic varices. Liver was normal in size measuring 9.5 cm with smooth margins and homogenous parenchyma and portal vein measuring 0.3 cm. Hepatic artery and vein were also unremarkable. No free fluid could be visualized in the abdominal cavity. Ultrasound was repeated along with doppler to confirm portal vein blood flow, which was absent. Computed tomography (CT) of the abdomen with contrast was performed next, which showed CTPV (Figure 1-2). The scan also displayed perisplenic varices, gross splenomegaly, and portosystemic shunt between the inferior mesenteric artery and left renal vein. Splenorenal varices were also identified. Upper gastrointestinal endoscopy was done, which showed the varices of grade 2. Workup for prothrombotic states was carried out, which showed the deficiency of protein C as 46% (70% to 140%) and antithrombin III to be 70% (74% to 126%).

The patient was treated with oral iron supplementation for anemia on which his hemoglobin improved over a period of two months. Beta-blocker was given for grade 2 varices. Anti-coagulation therapy was planned to be initiated as the patient had a positive thrombophilic profile. However, due to the limited role of of the anti-coagulation therapy in chronic extra-hepatic portal venous obstruction, it was delayed until after the shunt procedure was performed. The patient was referred for transjugular intrahepatic portosystemic shunt (TIPS).

## Discussion

CTPV, also known as portal cavernoma, is a sequela of thrombosis in the portal vein that may cause the development of abundant collateral vessels around an obstructed portal vein or even intra-hepatically [10]. This may lead to a rise in portal pressure, which results in the formation of these vessels. The etiology for the obstruction and portal hypertension may be pre-, intra- or post-hepatic. It may be congenital or acquired. Although rare, it is more often found in children rather than in adults. The pathogenesis of CTPV, however, in children often remains obscured [11].

There is a wide range of spectrum with which the patient presents. Gastroesophageal bleeding secondary to varices and hematological abnormalities in the form of thrombocytopenia and anemia occur because of the development of collateral channels around the portal vein [12]. Several times, patients also present with splenomegaly. Our patient also presented with similar hematological impairments and massive splenomegaly of 15 cm, as picked up on the ultrasound. In addition, there was also a second-degree oesophageal varix but that was asymptomatic.

The duration of symptoms may have a direct impact on the histological and physiological aspects of the liver, and hence the presenting age of the patient may determine the possible outcomes of the therapy and even the prognosis of the disease [12]. With advancing age, the progression in the cavernous transformation is increased, leading to further structural and functional impairment not only in the liver but also in the portosystemic circulation [13]. Our patient presented at a relatively younger age of nine years. In a study conducted on 30 children with symptomatic CTPV, placement of the shunt to decrease portal pressure was seen to be more effective in younger children and with lesser complications post-intervention as compared to those in the older children [12].

CTPV is usually diagnosed on Doppler ultrasound and CT scan, on which portal flow and pressures give indications for the presence of CTPV. Doppler ultrasound of our patient revealed the absence of blood flow in the portal vein, while CT findings were consistent with that of CTPV and its complications viz perisplenic varices, splenomegaly, and portosystemic shunts between the inferior mesenteric artery and left renal vein.

Upper gastrointestinal endoscopy must always be done to find out any underlying esophageal varices even when the patient is asymptomatic for this as was observed in our patient [14]. He had no hematemesis, but endoscopy revealed a grade 2 esophageal varix.

The etiology of CTPV varies widely and must be sought whenever possible to address the underlying cause [15]. The coagulation profile done in our patient revealed a deficiency of protein C and anti-thrombin III. These may have contributed toward a hypercoagulable state of the body, causing portal vein obstruction. Determination of the etiology of CTPV may also give a direction toward the management plan to not only relieve the patient of the already developed complications but also to treat the primary cause of the pathology [16].

Treatment may depend on the cause of CTPV but is usually to decrease portal pressure and relieve the symptoms of portal hypertension. Anti-coagulants are less likely to be employed for the management of CTPV due to an increased susceptibility of the patient to bleed secondary to portal hypertension and hence, TIPS can be opted instead [17]. The cause of portal hypertension is mainly due to thrombosis and cavernous transformation of extrahepatic portion of the portal vein. While this may allow the use of TIPS to relieve symptoms, it is not without its own sequelae which may include hepatic encephalopathy and growth retardation. The most recent technique utilizes the use of selective shunts like Rex bypass shunts and is now being increasingly favored for portal hypertension in children [12].

Our patient was treated symptomatically for anemia and varices. Since we belong to a third world country, the facility of REX shunt remains unavailable in most of the health-care centers including ours and thus, the patient was referred for TIPS and then planned to be kept on anti-coagulants afterward for his hypercoagulable state.

## Conclusions

CTPV, although rare, must be considered in children who present with symptoms that require urgent intervention like ascites, hepatosplenomegaly, or upper gastrointestinal bleeding. The etiology of CTPV must be meticulously sought such as a hypercoagulablility secondary to protein C and antithrombin III deficiency such that timely intervention can be done.
